# Clustering of clinical and echocardiographic phenotypes of covid-19 patients

**DOI:** 10.1038/s41598-023-35449-1

**Published:** 2023-05-31

**Authors:** Eran Shpigelman, Aviram Hochstadt, Dan Coster, Ilan Merdler, Eihab Ghantous, Yishay Szekely, Yael Lichter, Philippe Taieb, Ariel Banai, Orly Sapir, Yoav Granot, Lior Lupu, Ariel Borohovitz, Sapir Sadon, Shmuel Banai, Ronen Rubinshtein, Yan Topilsky, Ron Shamir

**Affiliations:** 1 The Blavatnik School of Computer Science, Tel Aviv University, P.O. Box 39040, 6997801 Tel Aviv, Israel; 2 Department of Cardiology, Tel Aviv Sourasky Medical Center, Dafna St 5, Tel Aviv-Yafo, Israel; 3 Heart Institute, Edith Wolfson Medical Center, Ha-Lokhamim St 62, 5822012 Holon, Israel; 4 The Sackler School of Medicine, The Tel-Aviv University, Tel Aviv, Israel

**Keywords:** Medical research, Cardiovascular diseases, Viral infection, Data processing, Machine learning

## Abstract

We sought to divide COVID-19 patients into distinct phenotypical subgroups using echocardiography and clinical markers to elucidate the pathogenesis of the disease and its heterogeneous cardiac involvement. A total of 506 consecutive patients hospitalized with COVID-19 infection underwent complete evaluation, including echocardiography, at admission. A k-prototypes algorithm applied to patients' clinical and imaging data at admission partitioned the patients into four phenotypical clusters: Clusters 0 and 1 were younger and healthier, 2 and 3 were older with worse cardiac indexes, and clusters 1 and 3 had a stronger inflammatory response. The clusters manifested very distinct survival patterns (C-index for the Cox proportional hazard model 0.77), with survival best for cluster 0, intermediate for 1–2 and worst for 3. Interestingly, cluster 1 showed a harsher disease course than cluster 2 but with similar survival. Clusters obtained with echocardiography were more predictive of mortality than clusters obtained without echocardiography. Additionally, several echocardiography variables (E′ lat, E′ sept, E/e average) showed high discriminative power among the clusters. The results suggested that older infected males have a higher chance to deteriorate than older infected females. In conclusion, COVID-19 manifests differently for distinctive clusters of patients. These clusters reflect different disease manifestations and prognoses. Although including echocardiography improved the predictive power, its marginal contribution over clustering using clinical parameters only does not justify the burden of echocardiography data collection.

## Introduction

COVID‐19 infection disease severity ranges widely, from asymptomatic or mild, self‐limiting illness to severe progressive pneumonia, multiorgan failure, and death^1^.

In addition to respiratory manifestations, several somewhat specific complications have been shown to be associated with COVID-19 illness, including cardiac and cardiovascular complications^2^, thromboembolic complications^3^, neurologic complications^4^, and inflammatory manifestations^5^. As the clinical picture is quite variable, a question emerges regarding the factors that direct the disease in a specific course.

The case of cardiac involvement is considerably diverse. Although cardiac complications are common and are associated with increased mortality^6^, cardiac involvement is heterogeneous, including right ventricular (RV) dysfunction or dilatation, left ventricular (LV) diastolic dysfunction and systolic dysfunction (10%)^2^.

As patients’ baseline characteristics and disease manifestations vary, we hypothesized that different epitomes of disease manifestations may be identified. To identify those, we sought to use a strategy of machine learning-based clustering. Unsupervised discovery of subtypes of a single disease has been widely used in cardiology^7^, infectious disease^8^, and critical care medicine^9^ to find better pathologic explanations and improve current treatments for these conditions.

Although several trials have endeavored unsupervised clustering of COVID-19 illness^10,11^, these included no comprehensive data regarding cardiac performance. As the echocardiographic data of patients with COVID-19 illness are essential for elucidation of both pathogenesis and prognosis^12^, we find this addition imperative to an enhanced clustering of COVID-19 illness.

## Methods

Details about data acquisition and gathering have been specified previously^2^. In brief, we prospectively studied consecutive adult patients (aged ≥ 18 years) admitted between March 21, 2020, and September 16, 2020, to the Tel Aviv Medical Center due to COVID‐19 infection. All patients had a diagnosis of COVID‐19 infection confirmed by a positive reverse‐transcriptase polymerase chain reaction assay. Demographic data, comorbid conditions, medications, physical examination, laboratory, and ECG findings were systematically recorded. All patients underwent comprehensive transthoracic echocardiography within 48 h of admission as part of a predefined step‐by‐step protocol. Clinical and imaging data were collected prospectively. Mortality analysis started at the time of baseline echocardiographic examination and included in‐hospital mortality. Mortality was ascertained until the end of follow-up, beyond hospitalization and irrespective of discharge date, for all patients by telephone calls and was complete for all the patients.

### Ethics approval

Since data were evaluated retrospectively, pseudonymously and were solely obtained for treatment purposes, the ethics committee of the Tel Aviv Medical Center approved the study (institutional review board number 0196‐20‐TLV) and voided the requirement of informed consent. The research was performed in accordance with the Declaration of Helsinki.

### Echocardiography

Echocardiography was performed in a standard manner with the same equipment (CX 50; Philips Medical Systems, Bothell, WA) by cardiologists with expertise in echocardiographic recording and interpretation. In accordance with current guidelines^13^, the following measures were undertaken to minimize the risk of infection: (1) All echocardiographic studies were bedside studies performed at the designated COVID‐19 intensive care or internal ward units. (2) All echocardiographic examinations were performed with small, dedicated scanners because of their easier disinfection. (3) Echocardiographic scanners were set aside in each COVID‐19-designated ward to minimize the risk of infection spread. (4) Personal protection at the time of echocardiographic recordings included N‐95 respirator masks, fluid‐resistant gowns, gloves, head covers, and eye shields. (5) Electrocardiographic monitoring during imaging was omitted, and all measurements were performed offline to reduce exposure and contamination. LV diameters, ejection fraction, and mass were measured as recommended^14^. Measurements of mitral inflow included the peak early filling (E wave) and late diastolic filling (A wave) velocities, E/A ratio, and deceleration time of early filling velocity. Early diastolic mitral septal and lateral annular velocities (e′) were measured in the apical 4‐chamber view^15^. Left atrial volume was calculated with the biplane area‐length method at end systole. Forward stroke volume was calculated from the LV outflow tract with subsequent calculation of cardiac output.

From 4‐chamber views encompassing the entire RV, end‐systolic and end‐diastolic RV areas and tricuspid annulus were measured. RV function was evaluated by tricuspid annular plane systolic excursion (TAPSE), systolic tricuspid lateral annular velocity measured in the apical 4‐chamber view, and fractional area change^14,16^. Hemodynamic right‐sided assessment included the measurement of the pulmonic flow acceleration time to assess pulmonary vascular resistance^17^.

Patients underwent another comprehensive echocardiographic test whenever there was any clinical deterioration, i.e., the need for mechanical ventilation and hemodynamic support, according to the treating physician’s judgment. This test was performed in the same manner as the first test performed upon arrival.

### The cohort

Patients who signed the DNR/DNI (n = 24) were removed from the cohort, resulting in 506 patients. For each patient, we used as input for clustering the measurements that were taken at admission and the first echocardiography result. Variables describing outcomes and treatments (n = 23) and medications (n = 43) were not part of the input data for clustering and were later used for evaluation of the clinical significance of the clusters. Variables that were missing in more than 2/3 of the cohort were also excluded (n = 23). We allowed a relatively high missing rate, as the data are sparse, to keep a high number of variables. The missing rate of each variable is found in Supplementary 3. This process left 85 continuous variables and 56 categorical variables. Thirty-one of the continuous variables and two of the categorical variables were from echocardiography.

### Computational methods

#### Imputation and normalization

Missing values in continuous variables were imputed using the Iterative Imputer algorithm based on MICE^18^. The continuous variables were normalized using the Yeo-Johnson power transform for nonnegative variables^19^ (see Supplementary 1). For the categorical variables, missing values were imputed with the most frequent value (another method for imputing the categorical variables achieved similar results. For details, see Supplementary 1).

#### Clustering

We used the k-prototypes^20^ algorithm, which is a distance-based algorithm that allows the use of mixed data, i.e., both categorical and continuous variables. We chose k-prototypes because it was reported as one of the best performers in a recent benchmark study on mixed-data clustering algorithms^21^. The algorithm receives as input the number of clusters $k$ and uses the distance function between variable vectors $x,y$:

1 $$d\left(x,y\right)= \sum_{j=1}^{p}{\left({x}_{j}-{y}_{j}\right)}^{2}+\gamma \sum_{j=p+1}^{m}\delta \left({x}_{j},{y}_{j}\right)$$

where ${x}_{1}, \dots {x}_{p}$ are numerical variables, ${x}_{p+1}, \dots {x}_{m}$ are categorical variables and $\delta$ is the Hamming distance function. $\gamma$ defines the relative weight assigned to the categorical variables. We used $\gamma =3$ and $k=4$ (see Supplementary 2, 3).

To obtain robust clustering, we applied consensus clustering^22^ to multiple clustering results obtained on subsampled data. In each repetition, we randomly chose a fraction $r$ of the patients and clustered them using k-prototypes. The process was repeated $n$ times. For patients $i$ and $j$, define $M(i,j)$ as the fraction of times in which they were in the same cluster, and the distance between them as $D(i,j)=1-M(i,j)$. Applying (regular) k-means on the distance matrix $D$ gives the final clusters. We used $r=0.85$ and $n=50$ (see Supplementary 2).

#### Evaluation of clusters

To evaluate the quality of the clusters, we looked for clinically significant characteristics of the patients composing the different clusters. For each variable, we tested its significance under the null assumption that it does not vary between clusters. For the continuous variables, we performed ANOVA, and for the categorical variables, we performed the Chi2 test, both implemented in SciPy^23^. P-values were corrected for multiple testing with FDR.

To find variables that are most discriminative between clusters, we computed the absolute standardized mean differences (ASMD) score (see Supplementary 1).

To visualize the different characteristics of the clusters, we created a radar plot of selected variables. Each variable was normalized to [0.2–1], where 0.2 is the lowest cluster average or percentage and 1 is the highest.

To analyze the survival trends in different clusters, we plotted the Kaplan–Meier survival curves^24^ for each cluster and computed the conditional multivariate log-rank test to compare the survival plots across the clusters. To measure the predictability of the clusters for in-hospital mortality, we estimated survival times from the Cox proportional hazard model^25^ with binary variables that represent the cluster’s membership as the covariates of the model and calculated the c-index^26^.

We also wished to evaluate whether the clusters showed distinct survival patterns among the patients who received respiratory support. For that, we built another Cox proportional hazard model for in-hospital mortality using only the patients who received respiratory support, with cluster labels as the covariates. We excluded Cluster 0, where only one patient received such support, and computed hazard ratios per cluster (relative to cluster 1). Implementations of Kaplan–Meier, Cox proportional hazard, C-index and log-rank were taken from lifelines^27^.

#### Evaluating the contribution of echocardiography

To evaluate the contribution of the 33 echocardiography variables to creating meaningful clusters, we tested the change in c-index obtained after randomly permuting across patients the values for each of the 33 variables, independently for each variable. In this way, we kept the distribution of each variable and the number of input variables unchanged. Next, we performed a similar process where we randomly chose 31 continuous variables and 2 categorical variables (the same numbers as for the echocardiography variables) and permuted their values to compare the contribution of the echocardiography data to randomly chosen variables. We also tested the change in echocardiography measurements over time using the results of a second echocardiography that some of the patients underwent. Full details of this analysis are found in Supplementary 6.

## Results

The final cohort studied included 506 hospitalized patients with PCR-positive COVID-19 of average age 62.31 and 36.96% females (n = 187). Further demographic and clinical characteristics are shown in Table 1.

**Table 1:** Demographics and selected clinical features of the cohort.

Feature	Average ± STD/percentage
Number of individuals	506
Age	62.31 ± 17.30
Sex (female)	36.96%
Total days in hospital	9.28 ± 12.02
MEWS score at admission	4.66 ± 3.15
CRP at admission	85.27 ± 78.01
History of hypertension	45.45%
Diabetes	31.42%
Obesity	26.09%
Ischemic heart disease (IHD) or congestive heart failure (CHF)	19.96%
Chronic renal failure (CRF)	9.68%
Dementia/cognitive decline	6.92%
Liver disease	3.56%

*MEWS* COVID-19 Modified Early Warning Score for clinical deterioration^28^, *CRP* C-reactive protein, a marker of inflammation.

### Identifying distinct patient subgroups

We clustered the patients into four clusters labeled 0 to 3, with sizes of 128, 195, 112, and 71, respectively. We analyzed several possible values for the number $k$ of clusters and selected k = 4 as the appropriate number (see full analysis in Supplementary 2). As we demonstrate below, the echocardiography parameters had a major contribution to the quality of the results.

The main parameters that significantly distinguish between the clusters are presented in Table 2, and the full list is shown in Supplementary 3. Based on these parameters, the most prominent characteristics of the clusters are as follows:

- **Cluster 0**—young patients (average age: $43.63$) with less medical history of significant diseases, with mild signs of inflammation at admission (low CRP) and no deaths.
- **Cluster 1**—relatively young patients (average age: $$61.27)$$ with a more severe inflammatory process. Of these, 27% received respiratory support, and 10% died while hospitalized.
- **Cluster 2**—older patients (average age: $$76.67)$$ with fewer inflammatory markers upon admission, with a richer medical history (e.g., 75% with hypertension) and more females than any other cluster (69%).
- **Cluster 3**—older patients (average age: 76.22) with marked inflammatory processes and a significant medical history (e.g., 83% with hypertension). These patients had the highest in-hospital mortality rate (54%).

**Table 2:** Statistics of selected variables that were significantly different among the clusters.

	Cluster 0	Cluster 1	Cluster 2	Cluster 3	p-value
Number of individuals	128	128	112	71	–
Age*	43.63 ± 12.82	43.63 ± 12.82	76.67 ± 10.88	76.22 ± 10.41	6 × 10^–82^
CRP*	33.71 ± 37.73	33.71 ± 37.73	36.31 ± 35.26	133.42 ± 81.62	6 × 10^–48^
MEWS score at Admission*	1.91 ± 2.07	1.91 ± 2.07	5.07 ± 2.46	7.68 ± 2.99	2 × 10^–32^
E/e average*	6.98 ± 1.59	6.98 ± 1.59	12.33 ± 5.56	14.35 ± 5.79	2 × 10^–35^
At*	108.43 ± 25.59	108.43 ± 25.59	81.26 ± 27.93	68.80 ± 19.75	3 × 10^–24^
O2 Saturation*	96.75 ± 3.13	96.75 ± 3.13	95.10 ± 4.77	86.45 ± 12.29	1 × 10^–17^
BNP	19.00 ± 17.85	19.00 ± 17.85	162.74 ± 206.94	609.94 ± 917.85	2 × 10^–16^
Troponin	8.49 ± 18.03	8.49 ± 18.03	20.84 ± 38.65	479.01 ± 1837.05	2 × 10^–3^
History of hypertension	9%	9%	75%	83%	7 × 10^–32^
Sex (Female)	38%	38%	69%	32%	3 × 10^–15^
Respiratory support**	1%	1%	13%	62%	1 × 10^–22^
In hospital mortality**	0%	0%	6%	54%	3 × 10^–27^

P-values were computed using ANOVA for continuous variables and Chi2 for categorical variables, and FDR corrected for multiple testing. *Continuous variables, mean ± SD of each cluster. **This outcome variables were not used by the clustering algorithm.

*CRP* C-reactive protein a marker of inflammation, *MEWS* COVID-19 Modified Early Warning Score for clinical deterioration^29^. *E/e average* a left ventricle echocardiography parameter, *At* a right ventricle echocardiography parameter.

**Figure 1:**
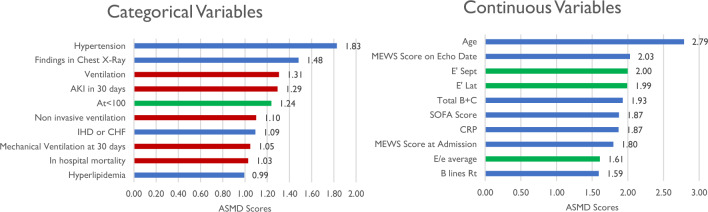
Variables with the highest ASMD scores. In red are outcomes that were not used for the clustering; in green are echocardiography variables. *Total B + C* a lung ultrasound parameter, combining consolidation and B line results.

To further assess the discriminative value of the different variables between the clusters, we calculated the absolute standardized mean differences (ASMD) scores for all variables. Figure 1 shows the variables with the highest scores. Among the variables with high ASMD are some natural COVID-19 parameters, such as CRP for inflammatory state, pulmonary ultrasound findings and findings in chest X-ray for lung function, and MEWS and SOFA, established warning scores for clinical deterioration. Age had the highest score, and several echocardiography variables, in particular left ventricle variables (E′ lat, E′ sept, E/e average), were scored high. We also evaluated the clinical merit of the clusters by calculating their discriminative power with respect to outcomes, which were not part of the input for clustering. Among them, in-hospital mortality and respiratory support-related variables scored high.

**Figure 2:**
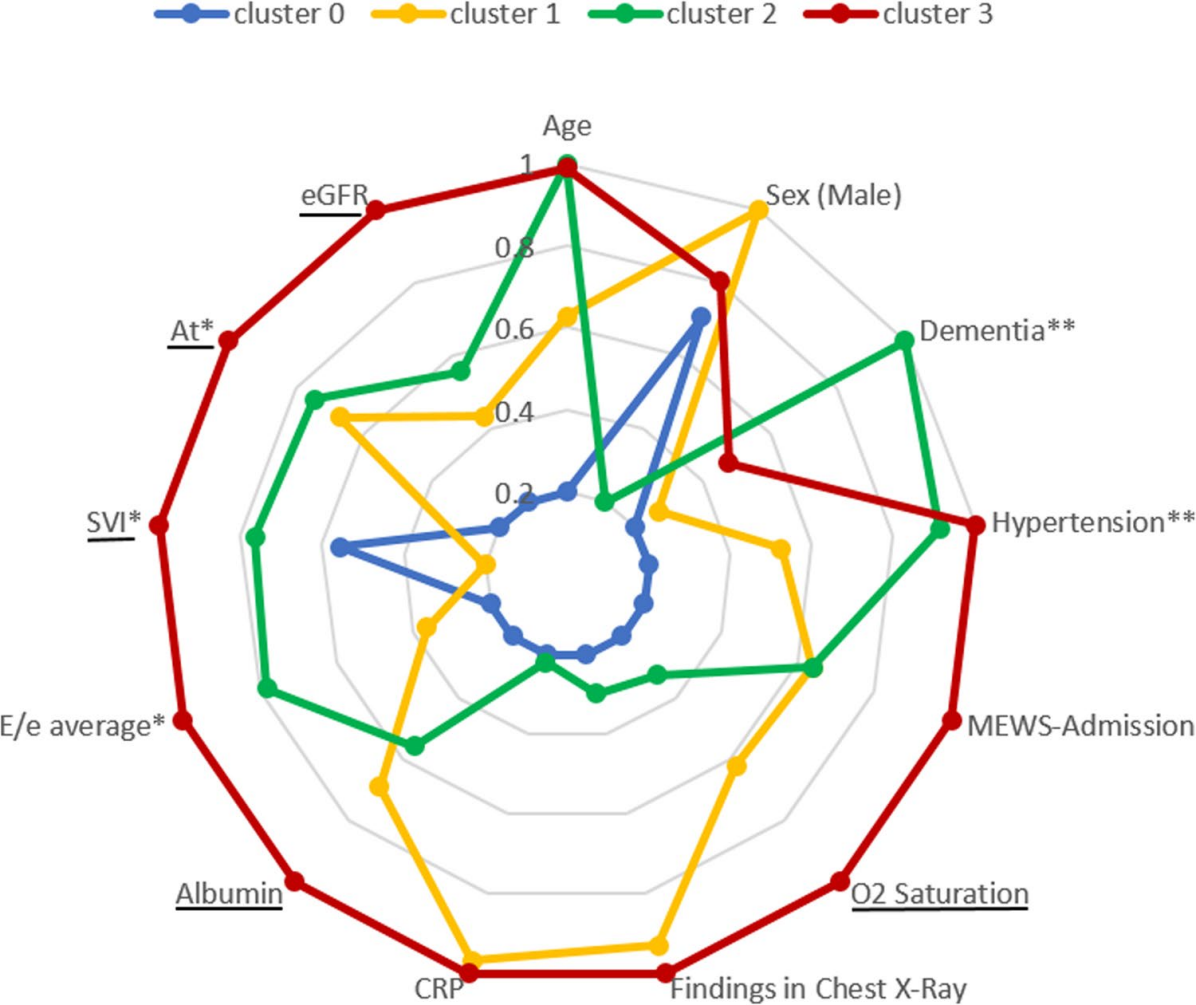
Radar plot for selected variables in clusters. Each variable is normalized to [0.2, 1], where 0.2 is the lowest cluster average/percentage and 1 is the highest. For ease of interpretability, the scale of some variables was reversed so that an increase from 0.2 to 1 always accounts for worse conditions. Underlined variables were reversed. *Echocardiography features: *E/e average* left ventricle feature, *At* right ventricle feature, *SVI* Stroke volume index. **Hypertension and *Dementia* are shown as examples for past diseases. Other past diseases showed similar trends (Supplementary 3). *CRP* C-reactive protein a marker of inflammation; *MEWS* COVID-19 Modified Early Warning Score for clinical deterioration^28^, *eGFR* Estimated Glomerular Filtration Rate.

Figure 2 shows a radar plot of significantly distinguishing variables (see Supplementary 4) that were selected to represent different aspects of the disease and the characteristics of the patients. Cluster 0 stands out as having the best results in most health-related parameters. In contrast, cluster 3 has the most severe results in most parameters. Cluster 2 patients have very similar age distributions to those in cluster 3, they have higher rates of dementia, and in both clusters we see high rates of hypertension and other comorbidities (likely due to high average age). However, remarkably, the COVID-19-related parameters of cluster 2 are much better: low CRP and high O2 saturation (this variable was reversed, so a high value in the plot means low O2 saturation). On the other hand, patients in cluster 1 were younger with fewer background diseases but worse COVID-19-related variables, such as high CRP, chest X-ray findings and O2 saturation.

Interestingly, sex significantly differed among the clusters. Cluster 2 is significantly enriched with female patients and the only cluster with a majority of females, while clusters 1 and 3 have a percentage of females below the cohort average (37%). A comparison of outcomes between males and females aged 80 and above in the full cohort showed that males are significantly more likely to receive respiratory support (p-value = 0.02), which is a sign for deterioration. All other outcomes were worse in males but not significantly so, perhaps due to the small sample size. For full details see Supplementary 7.

**Figure 3:**
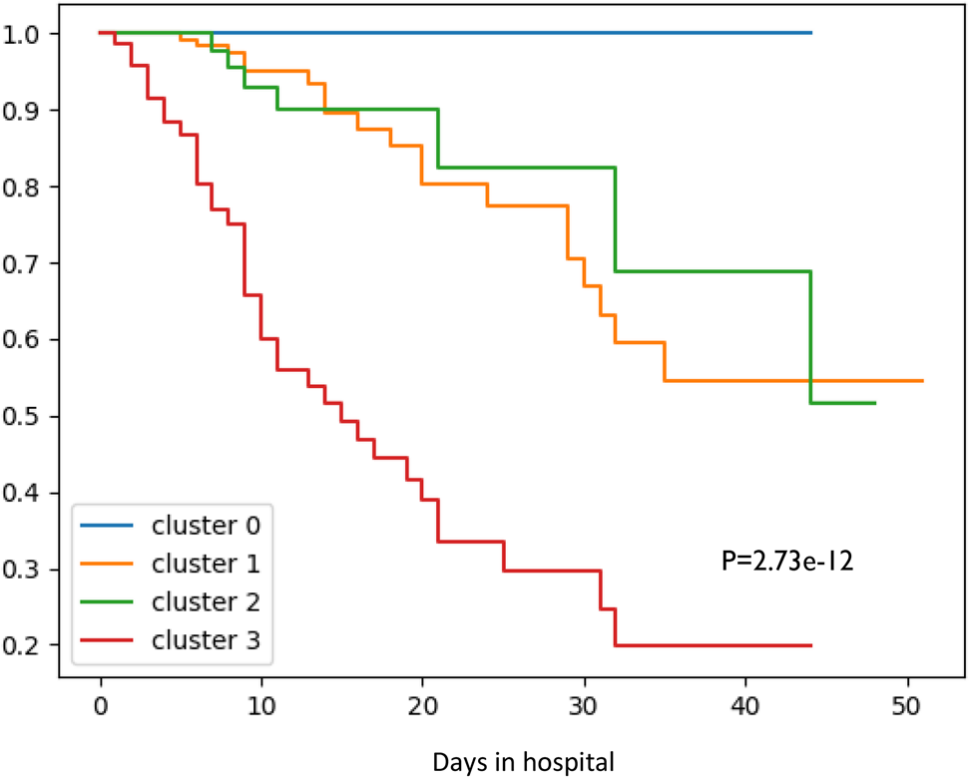
Kaplan–Meier survival curves for each cluster for the event “In-hospital mortality”. P is the log-rank p-value.

We also wished to evaluate to what extent the clusters differ in terms of in-hospital mortality. Figure 3 shows the Kaplan-Meyer survival curves of the four subgroups. We can see three distinct survival patterns, with no deaths in cluster 0, an intermediate survival pattern for clusters 1 and 2, and the worst survival of cluster 3. The log-rank test to assess differences in survival functions across clusters produced a highly significant p-value of 2.73e−12. The c-index for the Cox proportional hazard model (see “Methods”) was 0.77, which is relatively high.

Note that clusters 1 and 2 are very different in terms of age but have similar survival plots. For a detailed comparison of clusters 1 and 2, see Supplementary 5. While many parameters seem to be age related, Fig. 3 suggests that the course of the disease is not entirely dominated by age.

**Figure 4:**
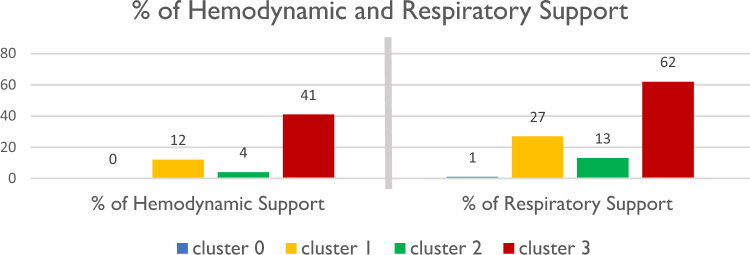
Percentage of patients who received respiratory support and hemodynamic support in each cluster.

Figure 4 shows the fraction of patients in each cluster who received respiratory and hemodynamic support. Patients in Clusters 1 and 3 suffered from severe disease, and therefore, a higher percentage of them were treated with respiratory or hemodynamic support (drug or mechanical).

Clusters 1–3 included a substantial number of patients who needed respiratory support. To test if their risk differed across clusters, we built a Cox proportional hazard ratio model for in-hospital mortality based only on those patients, using cluster labels as the covariates. Cluster 0 was excluded because only one patient in that group was ventilated. Cluster 1 was used as a reference. The hazard ratios were 1.25 (0.50, 3.14) for cluster 2 and 4.27 (2.42, 7.52) for cluster 3 (95% confidence interval in parentheses). Hence, ventilated patients in cluster 3 were at higher risk than those in cluster 1, although their inflammatory condition was similar at admission (CRP 129 vs. 133, see Table 1). Ventilated patients in cluster 2 were at a similar risk to those in cluster 1 despite the age difference between the groups. Although patients in cluster 2 were relatively older, they arrived in better inflammatory condition. The Cox model for the ventilated patients shows similar trends in survival to the full cohort, as was observed in the Kaplan–Meier curves in Fig. 3 (results not shown).

There were no significant differences between clusters in terms of treatment. For more details see Supplementary 8.

### Echocardiography contribution

Full statistics of the echocardiography variables are presented in Table 3. To further assess the contribution of the echocardiography data to the formation of meaningful clusters, we shuffled the echocardiography values, reclustered the resulting data 50 times (see “Methods”) and recomputed the c-index and log-rank p-values in each case. The average log-rank p-value was 1.97e−06 ± 6.54e−06 with a median of 3.98e−07, far less significant than on the original data (2.73e−12, Fig. 3). The average c-index was 0.74 ± 0.01, a decline of 0.03 compared to the initial clustering with the echocardiography variables. A clustering solution obtained without the echocardiography variables obtained a c-index of 0.74 ± 0.01 and a log rank p-value of 4.66e−06 ± 5.2e−0.06. While the gaps are modest, they are statistically significant. Together, these tests suggest that clusters obtained with echocardiography data are more predictive of mortality.

**Table 3:** Statistics of all echocardiography variables.

Variable	Cluster 0		Cluster 1		Cluster 2		Cluster 3		p-value
	mean ± std/percentage	# of patients	mean ± std/percentage	# of patients	mean ± std/percentage	# of patients	mean ± std/percentage	# of patients	
*E' Lat*	11.32 ± 2.76	126	8.82 ± 2.61	172	6.50 ± 2.01	110	6.55 ± 1.73	57	1.16E-45
*E' Sept*	8.37 ± 1.83	125	6.92 ± 1.68	171	5.37 ± 1.35	110	5.06 ± 1.16	58	2.68E-45
*E/e average*	6.98 ± 1.59	126	8.50 ± 3.01	170	12.33 ± 5.56	111	14.35 ± 5.79	58	1.87E-35
*E/E' Sept*	8.02 ± 1.89	115	9.34 ± 3.09	165	13.15 ± 6.39	104	16.21 ± 6.88	50	3.54E-29
*E/E' Lat*	6.01 ± 1.58	116	7.62 ± 3.32	164	11.28 ± 5.32	104	12.47 ± 5.33	49	1.02E-28
*A*	49.99 ± 10.23	125	62.76 ± 17.22	173	76.31 ± 20.72	98	69.13 ± 20.06	45	1.35E-25
*At*	108.43 ± 25.59	117	85.08 ± 21.33	162	81.26 ± 27.93	95	68.80 ± 19.75	60	3.21E-24
*IVSD*	7.51 ± 1.90	127	9.08 ± 2.06	176	10.24 ± 2.16	111	9.83 ± 2.29	62	1.89E-21
*Diastolic Grade*	0.21 ± 0.43	117	0.77 ± 0.99	152	1.41 ± 1.39	93	1.83 ± 1.46	41	9.78E-20
*RA Pressure*	6.09 ± 2.17	124	7.11 ± 3.09	161	8.19 ± 3.74	105	11.12 ± 4.69	58	1.45E-18
*E/A*	1.35 ± 0.40	125	1.03 ± 0.32	173	0.87 ± 0.26	98	1.20 ± 0.67	45	1.95E-18
**At < 100**	33%(38)	115	77%(123)	160	77%(71)	92	93%(52)	56	2.45E-18
*LV mass*	111.06 ± 36.50	125	151.03 ± 51.31	169	140.04 ± 50.25	108	171.47 ± 67.81	61	3.18E-14
*TAPSE*	2.39 ± 0.39	123	2.39 ± 0.48	170	2.15 ± 0.48	110	1.89 ± 0.47	61	2.55E-13
*LA volume*	46.23 ± 17.74	123	59.35 ± 24.03	172	61.43 ± 27.22	109	76.39 ± 29.07	61	4.84E-13
*EF*	58.58 ± 4.67	127	57.51 ± 5.39	174	58.21 ± 5.85	112	51.44 ± 9.52	59	1.27E-12
*LAVI*	24.61 ± 9.10	93	30.13 ± 11.43	139	34.86 ± 15.67	87	42.07 ± 17.33	46	2.36E-12
*SV*	60.28 ± 17.70	126	68.07 ± 16.25	174	54.61 ± 18.06	109	54.05 ± 16.66	62	7.02E-11
*LVEDD*	43.74 ± 5.72	126	45.90 ± 5.53	177	40.83 ± 6.87	110	46.75 ± 8.23	62	7.62E-11
*RVED area*	19.89 ± 4.32	99	21.99 ± 4.58	143	18.82 ± 4.70	90	23.65 ± 5.39	56	1.04E-09
*LVESD*	27.89 ± 4.98	124	29.92 ± 5.81	175	26.77 ± 5.84	106	32.33 ± 9.63	62	9.95E-08
*RV S’*	11.08 ± 1.94	122	12.03 ± 2.59	173	10.80 ± 3.12	104	9.73 ± 3.02	60	1.06E-07
Bad heart condition (≥ 2)*	1%(1)	128	2%(4)	195	11%(12)	112	18%(13)	71	2.12E-07
*RVES area*	11.27 ± 3.37	56	13.13 ± 3.97	66	10.67 ± 2.77	50	14.99 ± 5.80	31	6.30E-06
*E*	64.89 ± 14.21	127	62.52 ± 17.36	178	67.68 ± 25.86	112	77.82 ± 22.56	61	7.66E-06
*CO*	4.38 ± 1.22	126	5.69 ± 4.45	170	4.03 ± 1.28	107	4.23 ± 1.45	59	7.58E-06
*Pericardial fluid*	0.07 ± 0.26	127	0.08 ± 0.28	179	0.22 ± 0.42	112	0.30 ± 0.49	61	7.60E-06
*E decel time*	166.00 ± 35.04	115	180.34 ± 50.32	167	198.18 ± 56.85	102	165.64 ± 55.32	53	1.19E-05
*SVI*	32.47 ± 9.40	96	34.92 ± 8.70	141	31.04 ± 9.61	86	29.42 ± 9.82	47	1.34E-03
*HR at Echo date*	73.75 ± 12.35	126	78.92 ± 14.33	174	74.51 ± 15.09	108	80.00 ± 17.51	61	2.98E-03
*CI*	2.35 ± 0.66	96	3.00 ± 2.74	138	2.31 ± 0.76	84	2.26 ± 0.77	45	8.29E-03
*EF Simp*	65.10 ± 9.88	104	63.87 ± 12.43	149	63.63 ± 12.32	89	58.31 ± 15.68	49	1.87E-02
*RVFAC CALC*	45.63 ± 10.31	53	39.98 ± 13.56	64	42.53 ± 9.82	48	40.34 ± 12.56	29	7.16E-02

P-values were computed using ANOVA for continuous variables (in italics) and Chi2 for categorical variables (in bold), and FDR corrected for multiple testing. For the continuous variables mean ± SD values in each cluster are presented. *Bad heart condition (≥ 2) value of 2 or above of at least one of the following: AS, AR, MS, MR, TS, TR, PS, PR.

As another test of the contribution, we repeated the process of choosing at random 31 of the 85 continuous variables and 2 of the 56 categorical and shuffling their values. The average of 50 random choices was 0.73 ± 0.03, similar to just shuffling the echocardiography parameters. The average p-value of the log-rank test is 2.29e−04 ± 8.88e−04 with a median of 2.45e−08. This means that the echocardiography data are roughly as meaningful for forming the clustering as the rest of the parameters. It also shows redundancy in the contribution of different variables, as the changes are relatively small.

### Echocardiography over time

Forty-eight patients who suffered clinical deterioration underwent multiple echocardiography measurements adjacent to the time of deterioration (only the first echocardiography measurement was used as input for the clustering). The differences in selected echocardiography variables between the first and second echocardiography results are shown in Supplementary 6. Due to the small sample size, the differences are not statistically significant, but some parameters show a trend.

## Discussion

In the above trial, we have shown that hospitalized COVID-19 patients can be divided at admission into different clusters that have significant implications regarding disease course and final prognosis.

Although some aspects of the clusters seem self-evident (e.g., young patients have a better prognosis than old patients), others are less obvious, raising interest in the model’s ability to influence our understanding of COVID-19’s pathophysiology and progression and possibly help tailor treatments based on patient characteristics. The clusters significantly differed in natural COVID-19 parameters, such as CRP, chest X-ray findings and MEWS score, suggesting that the clusters are different in terms of the state of the disease. Moreover, the clusters are also separated by mortality- and respiratory-related variables that were not part of the input data, showing good separation among the clusters in terms of these outcomes. A further analysis of the survival patterns shows that the clusters manifest very distinct survival patterns.

The main point of interest is the difference between clusters 1 and 2. Patients in cluster 1 were younger with fewer chronic diseases and less cardiac involvement on the one hand and higher levels of inflammatory markers and markers of pulmonary involvement on the other hand. Cluster 2 had the highest average patient age, but lower levels of inflammatory markers and notably was the only cluster enriched with females. This suggests that older infected males may be more prone to deteriorate, in comparison to females in the same age group. This is also supported by significantly higher rate of respiratory support provided to older males, in comparison to older females, across the whole cohort. An interesting finding is that pulmonary artery acceleration time was similar between clusters, possibly signifying that it is a marker of both pulmonary dysfunction due to increased afterload because of pulmonary vasoconstriction and cardiac intrinsic dysfunction. Although these clusters had very different starting points on admission, the patient’s overall survival was similar in both. However, there were still significant differences in the need for hemodynamic support and respiratory support, with patients in cluster 1 necessitating more support. This difference might be explained by a different mechanism of deterioration between the two groups, possibly due to different pathological responses in the heart and lung systems between clusters. These differences show that the clusters are not only statistically and computationally justified but may also signify slightly different clinical entities.

Another noteworthy comparison is between cluster 1 and cluster 3. While the inflammatory state at admission was similar in the two clusters, the survival of cluster 3 was much worse, both when comparing the full clusters and when comparing only the ventilated patients. We can attribute the higher mortality to other factors, particularly age and comorbidity burden^28^.

### Variance in clinical deterioration of clusters

Another interesting finding is the difference between clusters of patients with clinical deterioration. Patients in clusters 1 and 3 had a decrease in stroke volume upon clinical deterioration together with an increase in right ventricular diastolic area (Table 3). This combination of an increase in RV preload (increase in right ventricular diastolic area) combined with a decrease in cardiac output (stroke volume) suggests that in patients in clusters 1 and 3, the right ventricle worked on the flat portion of the Frank–Starling curve^30^, signifying significant right ventricular failure. This is in contrast with patients from cluster 2 who had an increase in stroke volume with unchanged RV area, suggesting normal RV filling pressure and high output, indicating that in patients from cluster 2, clinical deterioration was due to cytokine storm and vasodilatation and not due to RV failure.

### Effect of echocardiography on clusters.

Although previous studies have tried to cluster COVID-19 patients, none have used echocardiography in the process. As previously demonstrated, echocardiography has added value over clinical characteristics alone in determining prognosis. In this study, we have shown that echocardiography data contributed to patient clustering in a modest but statistically significant manner and that clustering performed using echocardiographic data yields clusters that are better at predicting prognosis. However, conducting echocardiography is far more complex than obtaining the other clinical variables, the contribution of echocardiography, although significant, does not seem to justify the additional collection burden.

### Limitations

The study was performed only on patients who were admitted to the hospital and thus cannot be generalized to the entire population of patients with COVID-19. However, hospitalized patients were needed to perform echocardiography on each patient, and admitted patients are usually higher risk patients and thus of higher interest to study.

Furthermore, the study was performed on patients admitted during the first wave of COVID-19, before the emergence of new treatments, the availability of vaccines and the appearance of new strains of CVOID-19, such as the Delta and Omicron strains, and thus may not be relevant to the current manifestations of disease with newer treatments, vaccines and strains. Our group plans to perform a similar study on current patients to evaluate the clusters in a contemporary setting.

Additionally, this study was performed on patients admitted only to one center, and the results may not be generalizable to other cohorts of patients. Although we tried to find validation cohorts to compare the results and prevent overfitting, none were available that had comprehensive echocardiographic data. Furthermore, we used consensus clustering and carefully chose the clustering algorithm’s hyperparameters to ensure robust clustering results.

Regarding the echocardiography contribution, although the current results do not support performing echocardiography at baseline in all patients, we did not include direct cardiac complications (such as myocardial infarction, myocarditis, and cardiac arrhythmias) as outcomes. Therefore, we cannot estimate the predictive ability of baseline echocardiography and cluster assignment of these conditions.

In conclusion, hospitalized COVID-19 patients were segregated into different clusters according to demographic, clinical and echocardiographic data at admission. These clusters of patients showed different disease courses and proved valuable in determining prognosis.

## Supplementary Information

Supplementary Information.

## Data Availability

The data described and analyzed in this study may be made available upon reasonable request to Y.T. (yant@tlvmc.gov.il).

## Code availability

The code used in this study is available at https://github.com/Eranshp/clustering_echo_covid.git.
