# Supplementary material for: Clustering of clinical and echocardiographic phenotypes of covid-19 patients
Source: Sci Rep. 2023 May 31;13:8832. doi: 10.1038/s41598-023-35449-1 (PMC10231284; doi:10.1038/s41598-023-35449-1)
Supplement: Supplementary file 1 — Supplementary Information. [file 41598_2023_35449_MOESM1_ESM.pdf]

Supplementary material for:

CLUSTERING OF CLINICAL AND ECHOCARDIOGRAPHIC PHENOTYPES OF COVID-19 PATIENTS

Shpigelman E[1*], Hochstadt A[2,3*], Coster D[1], Merdler I[2], Ghantous E[2], Szekely Y[2], Lichter Y[2], Taieb P[2], Banai A[2], Sapir O[2], Granot Y[2], Lupu L[2], Borohovitz A[2], Sadon S[2], Banai S[2], Rubinshtein R[3] Topilsky Y[2], Shamir R[1]

[1] The Blavatnik School of Computer Science, Tel Aviv University, Tel-Aviv, Israel

[2] Department of Cardiology, Tel Aviv Sourasky Medical Center and Sackler School of Medicine, Tel Aviv University, Tel Aviv Israel

[3] Heart Institute, Edith Wolfson Medical Center, Holon, Israel and The Sackler School of Medicine, The Tel-Aviv University, Tel Aviv, Israel

[*]These authors contributed equally to this manuscript

Supplementary 1 – details of Methods

Yeo-Johnson transform - given a vector $X = (x_1, , , x_n)$ The Yeo-Johnson power transform for non-negative variables is defined as follows[1]:

$$\psi(\lambda, x_i) = \begin{cases} [(x_i + 1)^\lambda - 1]/\lambda & if\ \lambda \neq 0, x_i \geq 0 \\ \ln(x_i + 1) & if\ \lambda = 0, x_i \geq 0 \end{cases} \quad \text{(S1)}$$

λ is determined by maximizing the log likelihood function, with μ and σ^2 the mean and variance of the transformed observations, respectively:

$$l(\theta|x) = -\frac{n}{2}\log(2\pi) - \frac{n}{2}(\sigma^2) - \frac{1}{2\sigma^2}\sum_{i=1}^{n}\{\psi(\lambda, x_i) - \mu\}^2 + (\lambda - 1)\sum_{i=1}^{n} sgn(x_i)\log(|x_i| + 1) \quad \text{(S2)}$$

where $\theta = (\lambda, \mu, \sigma^2)'$ and $x = (x_1, \ldots x_n)'$.

We used Yeo-Johnson transform and Iterative Imputer (see computational methods) as implemented in Scikit learn[31].

Imputation of categorical variable– we imputed categorical variables in a relatively naïve approach of using the most frequent value. We also tested iterative imputation, which is a more advanced method, implemented in Scikit learn[2]. The clustering results obtained when applying the two imputation methods were very similar (Rand index 0.97 ± 0.01, adjusted Rand index of 0.92 ± 0.03), and we therefore decided to use the most frequent value for imputation.

Absolute standardized mean difference (ASMD) – Given a k-clustering of n vectors, consider a continuous variable v that has mean $\overline{V_i}$ and variance $S_{v_i}^2$ in cluster i for $i \in [1, \ldots, k]$. The ASMD of the variable[3,4] is defined as:

$$ASMD(v) = \frac{1}{\binom{k}{2}} \sum_{i,j\epsilon\{1..k),i<j} \frac{|\overline{V_i} - \overline{V_j}|}{\sqrt{\frac{S_{v_i}^2 + S_{v_j}^2}{2}}} \quad \text{(S3)}$$

In words, for every pair of clusters we calculate the standardized absolute difference between their means. The final score is the average over all the pairs.

Similarly, for a binary variable v:

$$ASMD(v) = \frac{1}{\binom{k}{2}} \sum_{i,j\epsilon\{1,..k\},i<j} \frac{\left|\hat{p}_{v_i} - \hat{p}_{v_j}\right|}{\sqrt{\frac{\hat{p}_{v_i}(1-\hat{p}_{v_i}) + \hat{p}_{v_j}(1-\hat{p}_{v_j})}{2}}} \tag{S4}$$

Where $\hat{p}_{v_i}$ is the fraction of vectors within category 1 for the variable in cluster i.

Supplementary 2: Choosing the number of clusters

To choose the number of clusters k, we ran the K-Prototypes algorithm for different values of k and computed the silhouette score[5] for each solution. We used K-Prototypes[6] and silhouette score[2] implementations. We clustered the patients based on the continuous variables only since they differed more significantly among subgroups (Table S8) and used the Euclidian distance. The results are shown in Figure S1.

We can see that solutions with 6 or more clusters give scores close to 0 or below, suggesting insignificant clusters. For four clusters the silhouette score was 0.052, and for five it was 0.030.

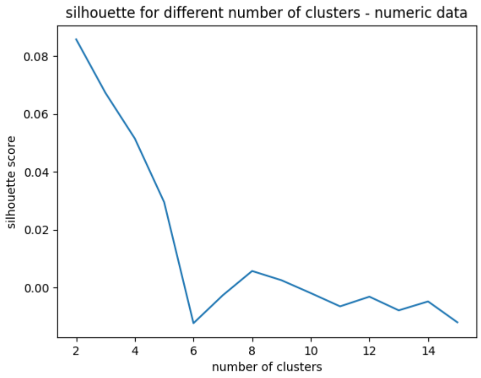

Figure S1. Change in silhouette score based on continuous variables for different number of clusters. The clustering algorithm we used was K-Prototypes with consensus clustering (50 iterations in each run, $= 3$, $r = 0.85$. details in supplementary 3).

For $2 \leq k \leq 5$ when using the "elbow method"[7,] $k = 4$ had a higher score than 3 and 5 (Figure S2). A solution with $k = 2$ had an even higher score, but we rejected that option since two clusters did not provide sufficient resolution and biomedical insights. Hence, we chose 4 as the final number of clusters.

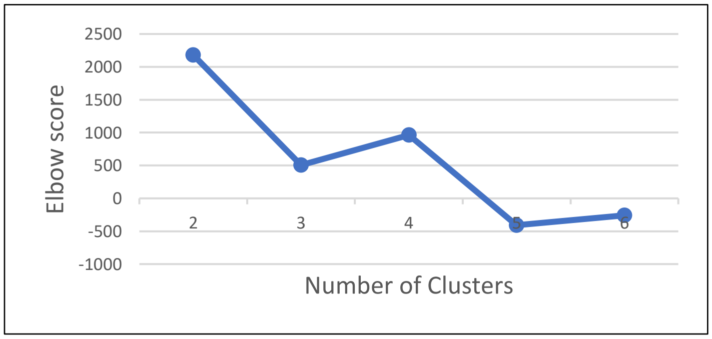

Figure S2. Scores to solutions with different number of clusters using the elbow method.

As an additional support for preferring four over three clusters, we focused on Clusters 1 and 2 in the solution with $k = 4$. The subgroups of patients in these two clusters had very similar survival curves (see Figure 3). We tested how distinct these two clusters are in terms of their other clinical parameters, and concluded that they were very distinct (Supplement 5). A solution with $k < 4$ does not show this distinction.

Supplementary 3 **Testing the Parameters of Consensus Clustering and K-Prototype**

To choose the sampling rate r in consensus clustering, and the relative weight γ assigned to categorial variables in K-prototypes, we ran the two algorithms for multiple combinations of parameters. For each combination, K-Prototypes was run 50 times with the consensus procedure. We used consensus clustering implementation [8]. Fig. S3 shows the average silhouette score for different values of γ and r. We can see that results for $r = 0.6$ were inferior, and for $\gamma = 3 - 5$, the solutions with $r = 1, 0.85, 0.75$ had similar scores. For $\gamma \geq 6$ the score for $r = 1$ was slightly higher. (In case $r = 1$ there is no sub-sampling, and minor differences between solutions are due to random initialization and cluster assignments in case of ties).

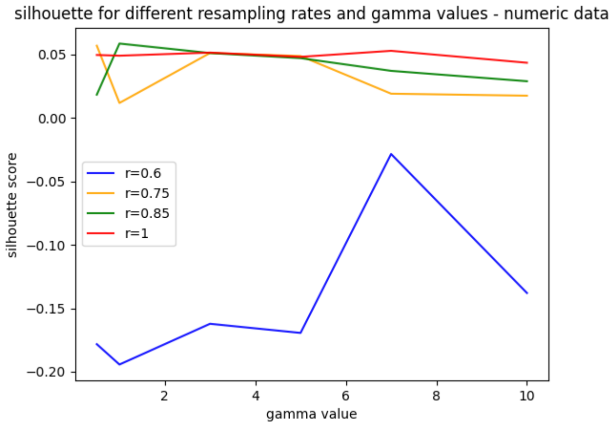

Figure S3. Silhouette score for a different r and γ values.

We wanted to make sure that the choice of γ guarantees that both the numerical and the categorical variables influence the clustering results. The default γ value used in the K-Prototype algorithm is half the average standard deviation of the numerical values. As we worked with normalized data, the default γ was 0.5. In tables S1-S12 we see the changes in the composition of the clusters in comparison to the clustering solution with the default γ. We can see that for $\gamma < 3$ the clusters do not change much, as also expressed by Rand index close to 1. This means that the categorical variables have very little effect on the solution for values close to the default γ. For $\gamma =$ 3-5 the categorical variables have more effect on the solution. For $\gamma > 5$ the structure of the clusters changes dramatically. Based on these observations and the silhouette scores, we chose $\gamma = 3$ and $r = 0.85$.

Table S1	$\gamma = 0.25$				
	Cluster number	0	1	2	3
Default γ	0	162	1	0	2
	1	0	142	0	0
	2	0	0	77	0
	3	0	0	0	122

Rand index: 0.99

Table S2	$\gamma = 0.5$				
	Cluster number	0	1	2	3
Default γ	0	164	0	0	0
	1	1	141	0	0
	2	1	0	76	0
	3	0	0	0	122

Rand index: 0.99

Table S3	$\gamma = 1$				
	Cluster number	0	1	2	3
Default γ	0	162	0	2	1
	1	5	136	0	1
	2	0	0	77	0
	3	1	3	0	118

Rand index: 0.95

Table S4	$\gamma = 3$				
	Cluster number	0	1	2	3
Default γ	0	156	1	7	1
	1	7	131	3	4
	2	10	0	65	2
	3	6	4	0	111

Rand index: 0.83

Table S5	$\gamma = 5$				
	Cluster number	0	1	2	3
Default γ	0	150	2	10	3
	1	16	121	0	5
	2	13	0	63	1
	3	17	2	8	95

Rand index: 0.73

Table S6	$\gamma = 7$				
	Cluster number	0	1	2	3
Default γ	0	100	54	7	4
	1	0	132	0	10
	2	17	0	57	3
	3	15	11	6	90

Rand index: 0.61

Tables S1-S6. The composition of the clusters for different values of γ compared to the default $\gamma = 0.5$. All runs used $r = 1$. The rows refer to the same four clusters obtained with the default γ, and the columns show clusters obtained for different values of γ, ordered for convenience so that the diagonal entries are maximal. Entries are the size of the intersection between the row and column clusters. For example, in table 1 a single patient moved from cluster 0 to 1, and in table 6 ten patients moved from cluster 1 to cluster 3. The Rand index quantifies the similarity between the two solutions, with 1 indicating a perfect match and 0 being the least possible. All runs used $r = 1$.

Table S7	$\gamma = 0.25$				
Default γ	Cluster number	0	1	2	3
	0	171	1	1	1
	1	0	139	0	0
	2	1	0	113	1
	3	1	0	0	77

Rand index: 0.99

Table S8	$\gamma = 0.5$				
	Cluster number	0	1	2	3
Default γ	0	115	0	0	0
	1	3	168	3	0
	2	0	0	139	0
	3	1	2	0	75

Rand index: 0.98

Table S9	$\gamma = 1$				
	Cluster number	0	1	2	3
Default γ	0	110	1	4	0
	1	1	170	1	2
	2	0	0	139	0
	3	0	2	0	76

Rand index: 0.98

Table S10	$\gamma = 3$				
Default γ	Cluster number	0	1	2	3
	0	101	13	0	1
	1	2	164	1	7
	2	6	8	125	0
	3	4	0	0	74

Rand index: 0.92

Table S11	$\gamma = 5$				
	Cluster number	0	1	2	3
Default γ	0	94	15	0	6
	1	4	154	2	14
	2	6	17	116	0
	3	2	14	0	62

Rand index: 0.85

Table S12	$\gamma = 7$				
	Cluster number	0	1	2	3
Default γ	0	92	15	2	6
	1	5	138	4	27
	2	8	15	116	0
	3	4	13	0	61

Rand index: 0.82

Tables S7-S12. The composition of the clusters for different values of γ compared to the default $\gamma = 0.5$. All runs used $r = 0.85$. More details in the description of Tables S1-S6.

Supplementary 4: **Distribution of all study variables across clusters**

The tables show the statistics for each variable that was included in the analysis. The mean ± standard deviation in each cluster are shown for continuous variables, and the fraction and number of patients in parentheses for categorical variables. The p-value measures the probability of obtaining the data for the variable under the null assumption that it does not vary between clusters. For the continuous variables we performed ANOVA test and for categorical variables we performed Chi2 test. P-values were FDR corrected for the multiple testing. In yellow are the continuous variables and in green are the categorical variables. Table S13: statistics for variables that were considered as input for the clustering. Table S14 shows parameters that were excluded from that process as they reflect data not available at the time of admission and initial tests.

Table S13: Variables used as input for the clustering

		Cluster 0		Cluster 1		Cluster 2		Cluster 3		
Variable	Missing Rate	mean±std/ percentage	# of patients	mean±std/ percentage	# of patients	mean±std/ percentage	# of patients	mean±std/ percentage	# of patients	p-value
Age	0.00	43.63 ± 12.82	128	61.27 ± 12.05	195	76.67 ± 10.88	112	76.22 ± 10.41	71	$5.63E-82$
CRP	0.02	33.71 ± 37.73	124	129.33 ± 76.69	193	36.31 ± 35.26	111	133.42 ± 81.62	69	$6.07E-48$
E' Lat*	0.08	11.32 ± 2.76	126	8.82 ± 2.61	172	6.50 ± 2.01	110	6.55 ± 1.73	57	$1.16E-45$
E' Sept*	0.08	8.37 ± 1.83	125	6.92 ± 1.68	171	5.37 ± 1.35	110	5.06 ± 1.16	58	$2.68E-45$
BUN	0.03	12.36 ± 4.43	121	18.48 ± 12.04	191	20.36 ± 10.28	111	44.14 ± 28.96	69	$8.73E-40$
MEWS Score on Echo Date	0.19	1.56 ± 1.88	101	5.11 ± 2.83	158	4.81 ± 2.60	99	8.29 ± 3.41	51	$7.50E-39$
E/e average*	0.08	6.98 ± 1.59	126	8.50 ± 3.01	170	12.33 ± 5.56	111	14.35 ± 5.79	58	$1.87E-35$
SOFA Score	0.44	0.32 ± 0.59	99	2.20 ± 2.49	84	1.06 ± 1.27	64	5.46 ± 3.06	37	$3.41E-33$
MEWS Score at Admission	0.21	1.91 ± 2.07	98	5.07 ± 2.83	151	5.07 ± 2.46	95	7.68 ± 2.99	56	$1.51E-32$

Hypertension	0.00	9%(11)	128	39%(76)	195	75%(84)	112	83%(59)	71	$6.65E-32$
eGFR - Creatinine	0.00	106.89 ± 31.15	128	89.96 ± 28.88	195	81.08 ± 33.19	112	49.00 ± 31.57	71	$5.67E-30$
E/E' Sept*	0.14	8.02 ± 1.89	115	9.34 ± 3.09	165	13.15 ± 6.39	104	16.21 ± 6.88	50	$3.54E-29$
E/E' Lat*	0.14	6.01 ± 1.58	116	7.62 ± 3.32	164	11.28 ± 5.32	104	12.47 ± 5.33	49	$1.02E-28$
Albumin	0.06	42.58 ± 3.79	112	37.68 ± 4.55	191	38.98 ± 4.71	106	34.57 ± 5.06	68	$3.47E-27$
A*	0.13	49.99 ± 10.23	125	62.76 ± 17.22	173	76.31 ± 20.72	98	69.13 ± 20.06	45	$1.35E-25$
At*	0.14	108.43 ± 25.59	117	85.08 ± 21.33	162	81.26 ± 27.93	95	68.80 ± 19.75	60	$3.21E-24$
Findings in Chest X-Ray	0.14	33%(37)	112	85%(137)	162	40%(41)	103	90%(54)	60	$5.01E-24$
Clinical Grade	0.44	1.00 ± 0.00	100	1.61 ± 0.76	84	1.08 ± 0.32	64	2.19 ± 1.10	37	$1.90E-23$
Fibrinogen	0.15	436.21 ± 121.82	90	604.98 ± 153.15	181	451.01 ± 113.07	94	532.87 ± 163.43	63	$5.18E-22$
IVSD*	0.06	7.51 ± 1.90	127	9.08 ± 2.06	176	10.24 ± 2.16	111	9.83 ± 2.29	62	$1.89E-21$
CRF	0.00	0%(0)	128	5%(9)	195	10%(11)	112	41%(29)	71	$2.44E-20$
IHD or CHF	0.00	3%(3)	128	11%(22)	195	32%(36)	112	55%(39)	71	$5.23E-20$
Diastolic Grade*	0.20	0.21 ± 0.43	117	0.77 ± 0.99	152	1.41 ± 1.39	93	1.83 ± 1.46	41	$9.78E-20$
Total B+C (a lung function parameter)	0.61	8.93 ± 4.62	57	17.32 ± 5.71	62	10.39 ± 5.92	49	19.10 ± 5.97	29	$1.20E-18$
RA Pressure*	0.11	6.09 ± 2.17	124	7.11 ± 3.09	161	8.19 ± 3.74	105	11.12 ± 4.69	58	$1.45E-18$
E/A*	0.13	1.35 ± 0.40	125	1.03 ± 0.32	173	0.87 ± 0.26	98	1.20 ± 0.67	45	$1.95E-18$
At<100*	0.16	33%(38)	115	77%(123)	160	77%(71)	92	93%(52)	56	$2.45E-18$
Fibrinogen on Echo Date	0.38	439.91 ± 124.11	69	614.94 ± 144.85	132	456.56 ± 111.84	66	539.26 ± 152.66	48	$2.47E-18$
Calcium	0.06	8.96 ± 0.48	112	8.53 ± 0.55	191	8.86 ± 0.60	106	8.25 ± 0.66	68	$1.10E-17$
O2 Saturation	0.12	96.75 ± 3.13	108	91.33 ± 7.86	181	95.10 ± 4.77	98	86.45 ± 12.29	56	$1.07E-17$
FiO2	0.44	21.14 ± 0.98	100	35.20 ± 24.23	84	22.47 ± 3.94	64	51.92 ± 33.66	37	$1.97E-17$
Hb	0.01	14.02 ± 1.45	126	13.67 ± 1.80	193	12.61 ± 1.85	112	11.78 ± 2.43	70	$2.41E-17$
Phospor	0.06	3.31 ± 0.60	112	3.19 ± 0.82	191	3.33 ± 0.84	106	4.41 ± 1.64	68	$9.36E-17$
BNP on Echo Date	0.37	20.12 ± 19.58	76	78.78 ± 106.54	119	159.83 ± 196.33	78	651.00 ± 960.42	46	$9.43E-17$
BNP	0.35	19.00 ± 17.85	82	78.12 ± 105.69	121	162.74 ± 206.94	77	609.94 ± 917.85	49	$2.13E-16$
Admission reason (12 categories)	0.09	–	127	–	164	–	107	–	62	$2.55E-16$
Sex (M=0, F=1)	0.00	38%(48)	128	20%(39)	195	69%(77)	112	32%(23)	71	$3.18E-15$
Height	0.22	1.71 ± 0.10	97	1.72 ± 0.08	157	1.62 ± 0.08	89	1.69 ± 0.07	52	$6.44E-15$
LV mass*	0.08	111.06 ± 36.50	125	151.03 ± 51.31	169	140.04 ± 50.25	108	171.47 ± 67.81	61	$3.18E-14$
Dementia/cognitive decline	0.00	0%(0)	128	2%(3)	195	23%(26)	112	8%(6)	71	$2.02E-13$
TAPSE*	0.08	2.39 ± 0.39	123	2.39 ± 0.48	170	2.15 ± 0.48	110	1.89 ± 0.47	61	$2.55E-13$
LA Volume*	0.08	46.23 ± 17.74	123	59.35 ± 24.03	172	61.43 ± 27.22	109	76.39 ± 29.07	61	$4.84E-13$
B lines Rt	0.61	2.40 ± 1.90	57	5.35 ± 2.16	62	3.27 ± 2.19	49	5.00 ± 1.98	29	$6.23E-13$
EF*	0.07	58.58 ± 4.67	127	57.51 ± 5.39	174	58.21 ± 5.85	112	51.44 ± 9.52	59	$1.27E-12$

LAVI*	0.28	24.61 ± 9.10	93	30.13 ± 11.43	139	34.86 ± 15.67	87	42.07 ± 17.33	46	$2.36E-12$
HCO3	0.20	25.21 ± 3.04	84	25.03 ± 3.25	178	25.97 ± 3.42	78	21.37 ± 5.80	66	$3.59E-12$
Afib/flutter	0.00	0%(0)	128	5%(10)	195	18%(20)	112	30%(21)	71	$8.18E-12$
Hyperlipidemia	0.00	10%(13)	128	34%(66)	195	54%(60)	112	45%(32)	71	$1.52E-11$
B lines Lt	0.61	2.79 ± 1.52	57	5.16 ± 2.05	62	3.35 ± 1.84	49	5.10 ± 2.09	29	$1.52E-11$
Ferritin	0.13	339.74 ± 362.08	104	1138.16 ± 1338.15	175	346.33 ± 376.99	100	1675.82 ± 2799.23	63	$2.08E-11$
Diabetes	0.00	9%(12)	128	32%(62)	195	40%(45)	112	56%(40)	71	$4.32E-11$
From nursing home/Institution	0.00	2%(2)	127	2%(4)	195	23%(26)	112	11%(8)	71	$4.44E-11$
SV*	0.07	60.28 ± 17.70	126	68.07 ± 16.25	174	54.61 ± 18.06	109	54.05 ± 16.66	62	$7.02E-11$
LVEDD*	0.06	43.74 ± 5.72	126	45.90 ± 5.53	177	40.83 ± 6.87	110	46.75 ± 8.23	62	$7.62E-11$
RVED Area*	0.23	19.89 ± 4.32	99	21.99 ± 4.58	143	18.82 ± 4.70	90	23.65 ± 5.39	56	$1.04E-09$
QTc	0.39	410.55 ± 25.27	64	425.77 ± 31.70	120	435.97 ± 30.43	77	451.65 ± 42.72	48	$1.40E-09$
Neutrophil	0.01	4.92 ± 4.89	126	6.09 ± 3.58	193	4.76 ± 2.51	112	8.61 ± 5.76	70	$3.22E-09$
Consolidation Rt	0.61	1.47 ± 1.80	57	3.15 ± 2.46	62	1.71 ± 2.29	49	4.34 ± 2.35	29	$3.85E-08$
TIA/CVA	0.00	1(1)	128	4%(7)	194	18%(20)	112	18%(13)	71	$4.91E-08$
LVESD*	0.08	27.89 ± 4.98	124	29.92 ± 5.81	175	26.77 ± 5.84	106	32.33 ± 9.63	62	$9.95E-08$
RV S'*	0.09	11.08 ± 1.94	122	12.03 ± 2.59	173	10.80 ± 3.12	104	9.73 ± 3.02	60	$1.06E-07$
Consolidation Lt	0.61	2.26 ± 1.90	57	3.82 ± 2.38	62	2.02 ± 2.16	49	4.66 ± 2.73	29	$1.90E-07$
Pleural Effusion Rt	0.61	0%(0)	57	0%(0)	62	2%(1)	49	24%(7)	29	$2.08E-07$
Weight	0.20	77.27 ± 17.98	99	84.70 ± 17.99	161	71.12 ± 15.52	90	77.55 ± 17.49	53	$2.13E-07$
bad heart condition (>=2)*	0.00	1%(1)	128	2%(4)	195	11%(12)	112	18%(13)	71	$2.12E-07$
AlkPhos	0.05	65.03 ± 25.88	114	72.76 ± 37.42	190	78.03 ± 42.90	107	103.13 ± 75.66	69	$6.06E-07$
SBP	0.02	128.77 ± 16.41	122	134.55 ± 19.12	194	143.70 ± 24.26	111	135.34 ± 24.24	70	$2.66E-06$
RVES Area*	0.60	11.27 ± 3.37	56	13.13 ± 3.97	66	10.67 ± 2.77	50	14.99 ± 5.80	31	$6.30E-06$
GGT	0.02	40.37 ± 42.01	124	82.06 ± 99.60	193	43.91 ± 70.17	110	89.61 ± 128.20	69	$7.03E-06$
PVD	0.00	1%(1)	128	1%(1)	195	7%(8)	112	13%(9)	71	$7.33E-06$
E*	0.06	64.89 ± 14.21	127	62.52 ± 17.36	178	67.68 ± 25.86	112	77.82 ± 22.56	61	$7.66E-06$
Glucose	0.01	105.00 ± 42.62	126	129.94 ± 64.81	193	122.08 ± 69.75	111	153.22 ± 71.73	69	$7.59E-06$
CO*	0.09	4.38 ± 1.22	126	5.69 ± 4.45	170	4.03 ± 1.28	107	4.23 ± 1.45	59	$7.58E-06$
Pericardial fluid*	0.05	0.07 ± 0.26	127	0.08 ± 0.28	179	0.22 ± 0.42	112	0.30 ± 0.49	61	$7.60E-06$
Temperature	0.01	37.38 ± 0.91	126	37.77 ± 0.84	193	37.40 ± 0.86	111	37.21 ± 1.04	70	$7.60E-06$
pH	0.20	7.37 ± 0.05	84	7.40 ± 0.06	178	7.38 ± 0.06	78	7.35 ± 0.11	66	$9.14E-06$
E decel time*	0.14	166.00 ± 35.04	115	180.34 ± 50.32	167	198.18 ± 56.85	102	165.64 ± 55.32	53	$1.19E-05$
Valvular Disease	0.00	1%(1)	128	2%(3)	195	7%(8)	112	14%(10)	71	$1.81E-05$
HR	0.02	85.14 ± 15.09	122	89.48 ± 14.24	194	80.05 ± 15.17	111	85.13 ± 21.01	70	$2.67E-05$

PCO2	0.20	45.18 ± 7.68	84	41.79 ± 8.33	178	44.74 ± 8.64	78	38.94 ± 10.48	66	2.88E − 05
DBP	0.02	79.21 ± 12.52	122	77.30 ± 12.17	194	75.60 ± 13.76	111	69.88 ± 14.35	70	3.74E − 05
D-Dimer on Echo Date	0.12	0.81 ± 1.32	111	1.77 ± 3.66	175	2.35 ± 4.85	97	3.60 ± 4.83	61	6.79E − 05
Afib/Aflutter	0.34	0%(0)	65	1%(2)	138	11%(9)	82	16%(8)	50	7.59E − 05
Pleural Effusion Lt	0.61	2%(1)	57	2%(1)	62	4%(2)	49	24%(7)	29	8.27E − 05
K	0.02	3.95 ± 0.40	126	3.97 ± 0.44	192	4.00 ± 0.49	110	4.26 ± 0.63	69	9.16E − 05
Troponin on Echo Date	0.25	9.23 ± 19.42	44	30.70 ± 130.83	168	28.96 ± 67.49	97	542.41 ± 1927.95	69	1.49E − 04
s/p CABG	0.00	0%(0)	128	2%(4)	195	4%(4)	112	11%(8)	71	2.43E − 04
D-Dimer	0.11	0.75 ± 1.23	112	1.90 ± 3.87	177	2.19 ± 4.70	100	3.11 ± 3.94	62	6.08E − 04
STD	0.34	0%(0)	64	1%(1)	136	1%(1)	82	10%(5)	50	6.90E − 04
NSR	0.34	97%(63)	65	99%(136)	138	89%(73)	82	84%(42)	50	7.82E − 04
Na	0.01	137.14 ± 2.48	126	135.28 ± 3.86	193	136.50 ± 3.88	111	136.16 ± 6.51	69	1.13E − 03
Subleural thick.	0.61	79%(45)	57	98%(61)	62	84%(41)	49	100%(29)	29	1.13E − 03
SVI*	0.27	32.47 ± 9.40	96	34.92 ± 8.70	141	31.04 ± 9.61	86	29.42 ± 9.82	47	1.34E − 03
WBC	0.01	7.14 ± 5.56	126	7.72 ± 3.96	193	6.77 ± 2.84	112	13.68 ± 31.41	70	1.50E − 03
Rate	0.37	80.33 ± 15.57	61	85.58 ± 16.00	132	77.18 ± 18.21	78	87.98 ± 23.32	49	1.69E − 03
Troponin	0.22	8.49 ± 18.03	49	62.15 ± 517.23	172	20.84 ± 38.65	103	479.01 ± 1837.05	70	2.20E − 03
HR at Echo date*	0.07	73.75 ± 12.35	126	78.92 ± 14.33	174	74.51 ± 15.09	108	80.00 ± 17.51	61	2.98E − 03
Thyroid Disease	0.00	5%(6)	128	6%(11)	195	15%(17)	112	15%(11)	71	3.13E − 03
Obesity	0.00	14%(18)	128	32%(63)	195	29%(33)	112	25%(18)	71	3.78E − 03
LDH	0.07	407.20 ± 140.24	111	786.37 ± 1583.42	187	444.92 ± 143.66	106	780.82 ± 601.19	67	3.84E − 03
BMI	0.22	26.26 ± 5.35	97	28.69 ± 5.25	157	27.07 ± 6.27	89	27.25 ± 5.54	52	8.15E − 03
CI*	0.28	2.35 ± 0.66	96	3.00 ± 2.74	138	2.31 ± 0.76	84	2.26 ± 0.77	45	8.29E − 03
CPK	0.07	153.39 ± 235.56	113	229.53 ± 352.20	188	124.17 ± 143.66	106	317.53 ± 794.50	66	8.71E − 03
L Axis	0.34	6%(4)	64	14%(19)	136	26%(21)	82	24%(12)	50	8.94E − 03
past or present Malignancy	0.00	7%(9)	128	12%(23)	195	21%(23)	112	20%(14)	71	9.64E − 03
Long QT	0.35	2%(1)	64	5%(6)	132	9%(7)	82	16%(8)	50	1.54E − 02
EF Simp*	0.23	65.10 ± 9.88	104	63.87 ± 12.43	149	63.63 ± 12.32	89	58.31 ± 15.68	49	1.87E − 02
LBBB	0.35	0%(0)	64	1%(1)	135	5%(4)	82	8%(4)	50	1.92E − 02
Pachy=0 Homogenous=1	0.62	155%(8)	55	0%(0)	61	13%(6)	48	0%(0)	29	3.02E − 02
Other Lung Diseases	0.00	1%(1)	128	7%(14)	195	7%(8)	112	10%(7)	71	3.95E − 02
COPD	0.00	2%(3)	128	6%(11)	195	7%(8)	112	13%(9)	71	4.15E − 02
Diffuse STE	0.34	3%(2)	64	0%(0)	136	0%(0)	82	0%(0)	50	4.78E − 02
Immune Deficiency	0.00	0%(0)	128	4%(7)	195	4%(5)	112	7%(5)	71	6.09E − 02
RVFAC CALC*	0.62	45.63 ± 10.31	53	39.98 ± 13.56	64	42.53 ± 9.82	48	40.34 ± 12.56	29	7.16E − 02

Inflammatory/Rheum Disease	0.00	2%(2)	128	9%(17)	195	7%(8)	112	6%(4)	71	$8.27E-02$
Psychiatric Disease	0.00	4%(5)	128	4%(8)	195	9%(10)	112	1%(1)	71	$1.11E-01$
Q inf	0.34	2%(1)	64	11%(15)	136	12%(10)	82	8%(4)	50	$1.31E-01$
3AVB	0.34	0%(0)	64	0.00 ± 0.00	137	0.00 ± 0.00	82	2.00 ± 1.00	50	$1.53E-01$
STE inf	0.34	0%(0)	64	0.00 ± 0.00	136	0.00 ± 0.00	82	2.00 ± 1.00	50	$1.53E-01$
Platelet	0.01	206.35 ± 81.62	126	203.44 ± 84.34	193	194.42 ± 79.01	112	224.14 ± 96.67	70	$1.67E-01$
Lymphocytes	0.02	1.52 ± 0.79	124	1.00 ± 0.95	193	1.30 ± 0.68	110	4.37 ± 28.78	68	$1.79E-01$
VTE	0.00	1%(1)	128	1%(2)	194	4%(4)	112	4%(3)	71	$1.86E-01$
Neurological Disease	0.00	2%(3)	128	4%(8)	195	8%(9)	112	7%(5)	71	$1.90E-01$
1AVB	0.34	0%(0)	64	4%(6)	137	4%(3)	82	8%(4)	50	$2.01E-01$
ALT	0.07	37.18 ± 31.00	120	75.12 ± 296.10	187	32.33 ± 68.50	102	37.49 ± 31.27	63	$2.01E-01$
R axis	0.34	0%(0)	64	1%(1)	136	4%(3)	82	2%(1)	50	$2.73E-01$
Bilirubin	0.02	0.57 ± 0.28	123	0.64 ± 0.57	193	0.52 ± 0.44	109	0.61 ± 0.82	69	$2.79E-01$
Q ant	0.35	0%(0)	64	2%(3)	135	5%(4)	82	4%(2)	50	$3.30E-01$
s/p ICD	0.00	0%(0)	128	0%(0)	195	1%(1)	112	0%(0)	71	$3.47E-01$
2AVB	0.34	0%(0)	64	1%(2)	137	0%(0)	82	0%(0)	50	$4.45E-01$
Q lat	0.35	0%(0)	64	4%(5)	135	4%(3)	82	2%(1)	50	$4.82E-01$
Liver Disease	0.00	2%(2)	128	4%(7)	195	5%(6)	112	4%(3)	71	$4.82E-01$
Asthma	0.00	5%(6)	128	5%(10)	195	8%(9)	112	3%(2)	71	$4.79E-01$
STE lat	0.34	0%(0)	64	1%(1)	136	0%(0)	82	2%(1)	50	$4.90E-01$
STE ant	0.34	0%(0)	64	2%(3)	136	1%(1)	82	0%(0)	50	$4.90E-01$
Smoking	0.06	9%(11)	123	6%(11)	183	11%(12)	108	9%(6)	64	$4.88E-01$
TWI	0.34	9%(6)	64	15%(20)	137	11%(9)	82	18%(9)	50	$5.06E-01$
AST	0.05	35.59 ± 20.78	116	106.50 ± 747.02	189	33.25 ± 32.62	107	67.24 ± 106.48	68	$5.07E-01$
NSIVCD	0.35	0%(0)	64	1%(2)	135	1%(1)	82	0%(0)	50	$6.62E-01$
RBBB	0.00	5%(3)	65	7%(10)	135	10%(8)	82	8%(4)	50	$7.12E-01$
Low voltage	0.02	0%(0)	64	1%(1)	136	1%(1)	82	2%(1)	50	$7.08E-01$

Table S13. Statistics per cluster of all variables that were included in the input for the clustering algorithm. P-values were computed using ANOVA for continuous variables and Chi2 for categorical variables, and FDR corrected for multiple testing. All variables refer to the first measurement taken at or after admission, unless otherwise noted. In yellow: Continuous variables, mean±SD in each cluster. In green: categorical variables, percentage (fraction of patients) for each cluster. *Echocardiography variables.

Table S14: Variables not used for the clustering

	Cluster 0		Cluster 1		Cluster 2		Cluster 3		
variable	mean±std/ percentage	# of patients	mean±std/ percentage	# of patients	mean±std/ percentage	# of patients	mean±std/ percentage	# of patients	p-value
In hospital mortality	0% (0)	128	10% (19)	195	6% (7)	112	54% (38)	71	3.04E-27
30 day mortality	0% (0)	128	9% (17)	195	4% (5)	112	51% (36)	71	1.83E-27
Ventilation	1% (1)	128	27% (52)	195	13% (14)	112	62% (44)	71	1.03E-22
AKI 30 days	2% (3)	128	29% (57)	195	21% (23)	112	68% (48)	71	5.85E-22
Mechanical Ventilation at 30 days (y/n)	1% (1)	128	17% (34)	195	6% (7)	112	49% (35)	71	1.68E-19
Hemodynamic Support	0% (0)	128	12% (24)	195	4% (5)	112	41% (29)	71	1.77E-17
Non invasive ventilation	0% (0)	128	23% (45)	195	10% (11)	112	45% (32)	71	1.20E-15
days in hospital	3.63±4.94	127	11.88±14.46	195	8.88±11.34	112	12.83±11.18	71	1.26E-09
Mechanical Ventilation	0% (0)	100	5% (5)	92	3% (2)	68	24% (9)	38	8.67E-07
Venous thromboembolism (VTE) 30 days	0% (0)	82	8% (10)	125	2% (3)	88	4% (2)	48	3.32E-02
Major bleed 30 days	0% (0)	99	2% (3)	129	1% (1)	92	6% (3)	48	7.07E-02
Stroke 30 days	0% (0)	82	2% (3)	126	0% (0)	87	2% (1)	47	2.64E-01

Table S14. Statistics per cluster of variables that were not part of input for the clustering algorithm. See Table S13 caption for additional details.

Supplementary 5: **Differences between clusters 1 and 2**

Clusters 1 and 2 had similar survival trends (see Results) but different characteristics of the patients comprising the clusters. Fig. S4 shows the top ASMD scored variables when comparing only these two clusters. They include variables that are directly related to covid-19 like CRP and findings in chest X-ray, alongside age and age-related variables like past diseases (hypertension, dementia, IHD or CHF) and arriving from nursing home. Table S15 lists the top 20 variables with the lowest p-value for the null assumption that they do not vary between the two clusters, with similar results to ASMD.

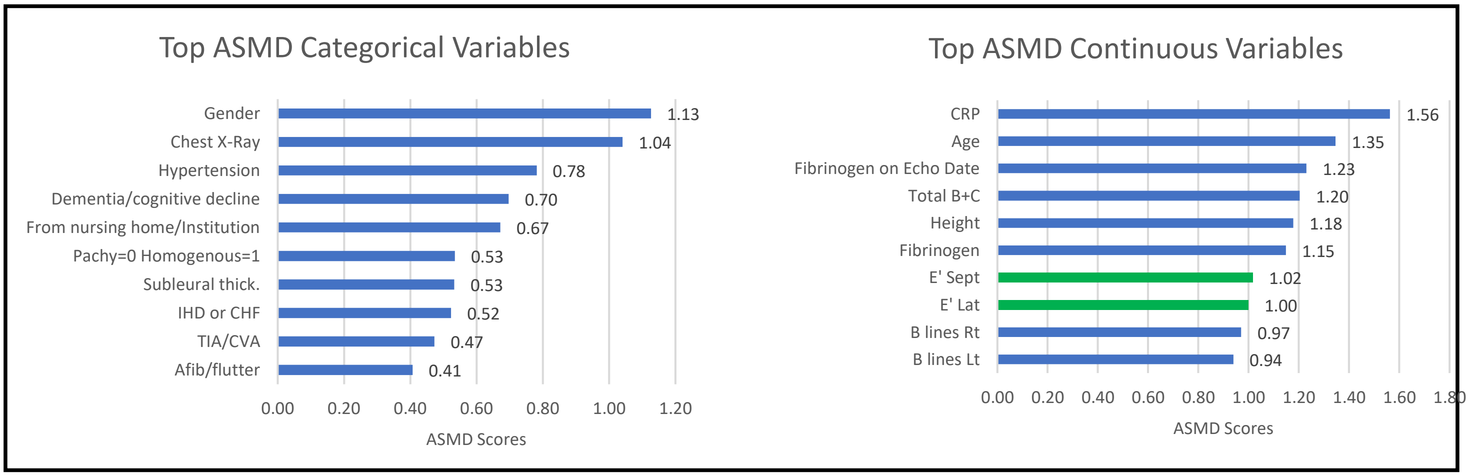

Figure S4. Variables with the highest ASMD scores when comparing clusters 1 and 2. In green – echocardiography variables.

Table S15: Parameters that distinguish most between clusters 1 and 2

	Cluster 1		Cluster 2		
variable	mean±std/ percentage	# of patients	mean±std/ percentage	# of patients	p-value
CRP	129.33±76.69	193	36.31±35.26	111	1.31E-27
Age	61.27±12.05	195	76.67±10.88	107	1.72E-24
Sex (M=0, F=1)	20%(39)	195	69%(77)	112	6.36E-17
Height	1.71±0.08	157	1.62±0.08	89	3.98E-16
Fibrinogen	604.98±153.15	181	451.01±113.07	94	6.23E-16
E' Sept*	6.92±1.68	171	5.37±1.35	110	1.72E-14
E' Lat*	8.82±2.61	172	6.50±2.01	110	5.35E-14
Findings in Chest X-Ray	85%(137)	162	40%(41)	103	1.09E-13
Fibrinogen on Echo Date	614.94±144.85	132	456.56±11.84	66	3.73E-13
E/e average*	8.50±3.01	170	12.33±5.56	111	1.09E-12
E/E' Lat*	7.62±3.32	164	11.28±5.32	104	3.08E-11
LVEDD*	45.90±5.53	177	40.83±6.87	110	4.04E-11
E/E' Sept*	9.34±3.09	165	13.15±6.39	104	3.13E-10
SV*	68.07±16.25	174	54.61±18.06	109	3.82E-10
Dementia/cognitive decline	2%(3)	195	23%(26)	112	1.46E-09
Hypertension	39%(76)	195	75%(84)	112	2.46E-09
From nursing home/Institution	2%(4)	195	23%(26)	112	6.18E-09
Weight	84.70±17.99	161	71.12±15.52	90	6.35E-09
Total B+C (a lung function parameter)	17.32±5.71	62	10.39±5.92	49	8.11E-09
Ferritin	1138.16±1338.15	175	346.33±376.99	100	2.00E-08

Table S15. Parameters that distinguish most between clusters 1 and 2. Shown are the 20 variables with lowest p-value. P-values were computed using t-test for continuous and Chi2 for categorical variables, and FDR corrected for multiple testing. All variables refer to the first measurement taken at or after admission, unless otherwise noted. In yellow: Continuous variables, mean±SD in each cluster. In green: categorical variables, percentage (num of patients) for each cluster. *Echocardiography variables.

Supplementary 6: **Echocardiography over time**

We tested the change in echocardiography measurements over time, using data of a second echocardiography that 48 of the patients underwent. We chose nine variables of interest and performed Kruskal–Wallis test on the differences between the second and the first echocardiography results in each cluster. We chose Kruskal–Wallis since the sample sizes were low. The p-values were corrected for multiple testing with FDR. The results are summarized in Table S16. While some trends can be observed between clusters, due to the small sample size the p-values are high.

Table S16 - The change in repeated echocardiography results for selected variables.

	Cluster 1		Cluster 2		Cluster 3			
	Mean change	#	Mean change	#	Mean change	#	p-value Kruskal–Wallis	FDR corrected p-value
SV	-11.93±23.78	23	8.67±17.24	6	-8.50±10.60	12	0.05	0.49
RVED Area	4.83±5.59	17	0.24±5.43	7	0.64±4.44	11	0.06	0.27
LA Volume	-6.66±19.03	18	11.46±13.77	8	0.60±39.71	13	0.09	0.28
E decel time	14.73±55.75	22	-41.50±58.80	8	-2.60±70.50	15	0.10	0.22
LVEDD	-1.13±8.45	25	0.63±7.77	7	-3.41±6.82	15	0.28	0.50
TAPSE	-0.32±0.44	18	-0.19±0.84	8	-0.33±0.46	15	0.36	0.54
At	-5.83±35.94	18	-21.20±30.46	5	-4.29±26.02	14	0.66	0.85
RA Pressure	2.65±5.89	17	3.33±6.06	6	2.92±4.98	12	0.96	1.00
E/E' Lat	-0.05±3.56	18	-0.27±4.88	8	-0.13±4.88	14	1.00	1.00

The values are the mean change between the second and the first echocardiography measurements, for patients who underwent two or more measurements during their hospitalization. Cluster 0 was excluded as it had only one patient with a second echocardiography. #: number of patients in each cluster. In green are the right ventricle parameters, and in yellow the left ventricle's.

Supplementary 7: **Evaluating Sex Differences**

The clusters suggested that older females may have better chance to experience only a mild disease. Therefore, we performed an analysis of the outcomes for patients aged 80+, comparing males and females. Males received significantly higher rates of respiratory support. They also had higher rates in all tested outcomes, but the differences were not significant (Table S17).

Table S17: Outcome analysis for males and females aged 80 and above.

	Males		Females		
variable	mean±std/ percentage	# of patients	mean±std/ percentage	# of patients	p-value
Ventilation	50% (18)	36	18% (9)	50	0.02
Hemodynamic Support	33% (12)	36	12% (6)	50	0.06
AKI in 30 days	53% (19)	36	30% (15)	50	0.07
30 day mortality	39% (14)	36	18% (9)	50	0.08
Mechanical ventilation	36% (13)	36	14% (7)	50	0.08
In hospital mortality	42% (15)	36	18% (9)	50	0.1
Days in hospital	10.19±8.23	36	9.06±11.32	50	0.6

Table S17. Outcomes analysis for males and females aged 80 and above. P-values were computed using t-test for continuous and Chi2 for categorical variables, and FDR corrected for multiple testing. Outcomes that were present in five patients or less were not considered. In yellow: Continuous variables, mean±SD in each cluster. In green: categorical variables, percentage (num of patients) for group.

Supplementary 8: **Treatments**

The data was gathered during the first months of the pandemic (March to September 2020), and before any targeted anti-viral effective treatments for Covid-19 were introduced to clinical practice. 37 patients were treated with Systemic corticosteroids and 24 patients were on Clexane. The medications do not add new insights about the clusters.

Clexane was admitted to 2% of patients in cluster 0 and 6% for each of the other clusters. Systemic corticosteroids were admitted to 1% of patients in cluster 0, 9% in cluster 1, 4% in cluster 2 and 15% in cluster 3, which aligns with the higher rates of inflammation in clusters 1 and 3.

References

1. Yeo I kwon, Johnson RA. A New Family of Power Transformations to Improve Normality or Symmetry. Vol 87.; 2000. https://academic.oup.com/biomet/article/87/4/954/232908

2. Pedregosa F, Michel V, Grisel O, et al. Scikit-learn: Machine Learning in Python. Journal of Machine Learning Research. 2011;12:2825-2830. http://scikit-learn.sourceforge.net.

3. Preud'homme G, Duarte K, Dalleau K, et al. Head-to-head comparison of clustering methods for heterogeneous data: a simulation-driven benchmark. Scientific Reports. 2021;11(1). doi:10.1038/s41598-021-83340-8

4. Austin PC. An introduction to propensity score methods for reducing the effects of confounding in observational studies. Multivariate Behavioral Research. 2011;46(3):399-424. doi:10.1080/00273171.2011.568786

5. Rousseeuw PJ. Silhouettes: A graphical aid to the interpretation and validation of cluster analysis. Journal of Computational and Applied Mathematics. 1987;20(C):53-65. doi:10.1016/0377-0427(87)90125-7

6. Nelis J. de Vos. kmodes categorical clustering library. Published online 2015.

7. Rappoport N, Shamir R. Multi-omic and multi-view clustering algorithms: review and cancer benchmark. Nucleic Acids Research. 2018;46(20):10546-10562. doi:10.1093/nar/gky889

8. X. Žiga Sajovic. consensus clustering. Published online 2019.
